# Acquired ALK G1202R-, ALK I1171N-, or EML4-ALK-mediated resistance to ensartinib in lung adenocarcinoma but responded to lorlatinib: A case report

**DOI:** 10.3389/fonc.2023.1082115

**Published:** 2023-03-16

**Authors:** Zhifeng Ye, Junhua Guo

**Affiliations:** Department of Oncology, Hangzhou Chinese Traditional Medicine Hospital, Hangzhou, China

**Keywords:** lung cancer, multiple ALK mutations, I1171N, ensartinib, lorlatinib

## Abstract

ALK rearrangements are identified as driver mutations in non-small-cell lung cancer (NSCLC). EML4 is the most common partner of ALK rearrangements. Here, we reported a patient with lung adenocarcinoma who was identified with EML4-ALK mutations when he progressed on an immune checkpoint inhibitor. The patient was treated with alectinib and obtained a progression-free survival (PFS) of 24 months. Then, next-generation sequencing on circulating tumor DNA identified multiple ALK mutations, including ALK G1202R, I1171N, ALK-ENC1, and EML4-ALK. Ensartinib was given, and the patient achieved a PFS of 5 months. After progression, lorlatinib was administered, and the patient achieved a partial response. Now, the benefit is still ongoing with a PFS over 10 months. Our case may provide evidence for the treatment choice of multiple ALK mutations, including ALK I1171N.

## 1 Introduction

Lung cancer is one of the most common causes of cancer-related death worldwide. Anaplastic lymphoma kinase (ALK) belongs to the family of tyrosine kinases involved in the pathogenesis and development of lung cancer (1). It has been well-documented that ALK fusions play an essential role in the oncogenic process of lung cancer by activating downstream signaling cascades, such as the PI3K/mTOR pathway (2). ALK gene consists of 30 exons mapping to the long arm of chromosome 2 (2p23.2–p23.1) (3). Approximately 3%–10% of non-small cell lung cancer (NSCLC) patients have ALK fusions (1, 4–7), with EML4-ALK fusion accounting for approximately 80% (7, 8).

Many previous studies show that ALK fusions responded well to ALK inhibitors. Nevertheless, several studies report that different ALK partners responded differently to ALK inhibitors (6). Even I1171N has been proposed to resist the second-generation ALK inhibitor alectinib (8).

Herein, we presented a patient with NSCLC harboring multiple ALK fusion mutations, including I1171N, who experienced rapid disease progression after ensartinib treatment but responded well to lorlatinib. This case provides a meaningful reference for treating NSCLC patients with ALK I1171N and other ALK fusions.

## 2 Case presentation

**Figure 1:**
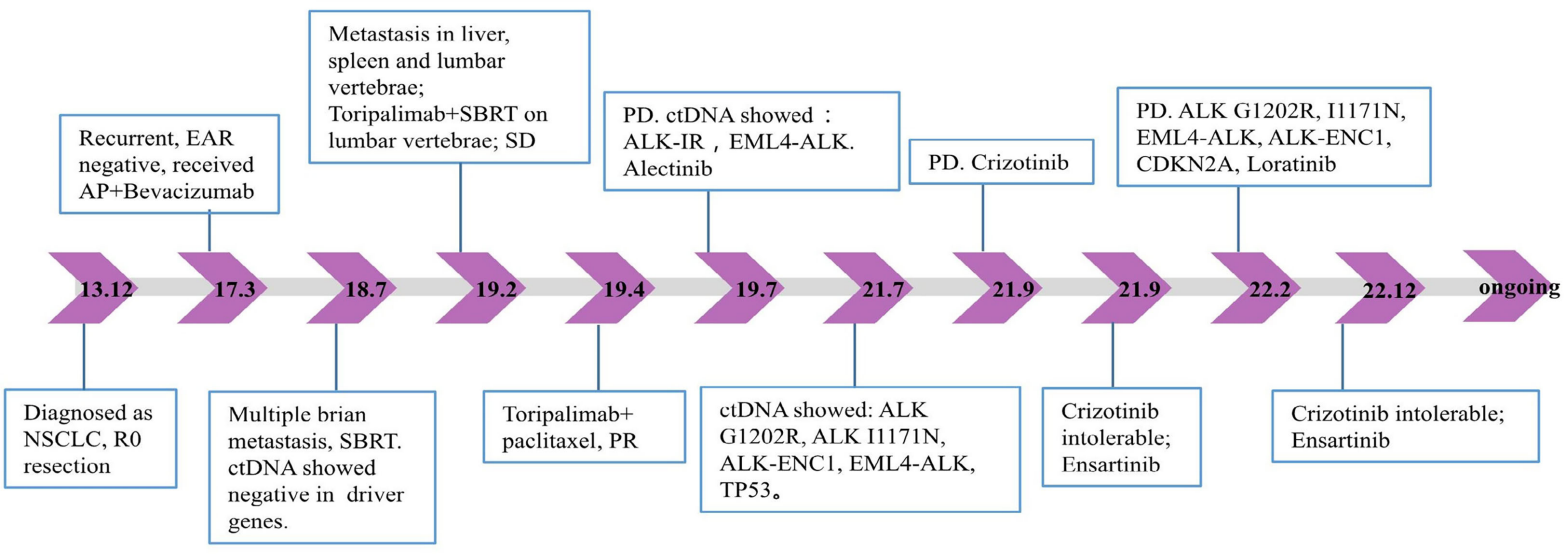
Timeline of the patient’s treatment history and plasma NGS results. EAR, EGFR, ALK, and ROS1; AP, pemetrexed plus cisplatin; NSCLC, non-small-cell lung cancer; SBRT, stereotactic body radiation therapy; SD, stable disease; ctDNA, circulating tumor DNA; PR, partial response; PD, progressive disease; NGS, next-generation sequencing.

**Figure 2:**
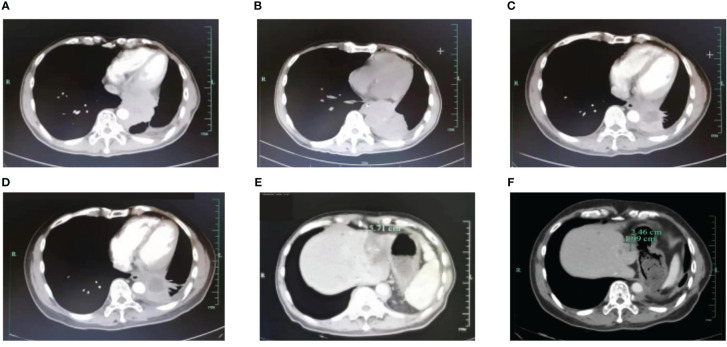
Radiographical changes. **(A)** PET/CT scan shows the failure of AP+Bevacizumab treatment in March 2019. **(B)** CT scan shows the failure of toripalimab+paclitaxel in July 2019. CT shows a durable response to alectinib in December 2019 **(C)** and 2020 **(D)**. **(E)** PD on ensartinib in February 2022. **(F)** CT shows a partial response to lorlatinib in December 2022. AP, pemetrexed plus cisplatin; PD, progressive disease; PET/CT, positron emission tomography/computed tomography.

A 53-year-old Asian man, who is a heavy smoker, was diagnosed with lung adenocarcinoma in December 2013 and subsequently underwent R0 resection (**Figure 1**). Unfortunately, recurrence occurred in March 2017. Intra-hospital detection of biopsy samples revealed negative EAR (EGFR, ALK, and ROS1). Then, bevacizumab combined with AP chemotherapy was applied, and the best response was partial response (PR). Multiple central nervous system (CNS) metastases were found after 16 months of treatment. The physical condition and the location of metastases made this patient not suitable for biopsy. Alternatively, non-invasive circulating tumor DNA (ctDNA) detection was performed using a nosocomial test and found to be negative in driver genes, such as EGFR, ALK, ROS1, and MET. Stereotactic body radiation therapy (SBRT) was applied to treat CNS metastases. In February 2019, metastases occurred in the liver, spleen, and lumbar vertebrae. Toripalimab and SBRT were started, and the best response was stable disease (SD). Then, toripalimab plus paclitaxel was initiated, achieving a PR at the first evaluation but developing into progressive disease (PD) at 3 months treatment (**Figures 2A, B**). Considering the rapid tumor growth and the urgent condition of the patient, we conducted the ctDNA next-generation sequencing (NGS) with a depth of 36,000× for additional treatment choices and identified the ALK-intergenic region (ALK-IR) and EML4-ALK mutations. According to the National Comprehensive Cancer Network (NCCN) guidelines, the patient received alectinib treatment in July 2019 (**Figures 2C, D**). Follow-up examination found an elevation of carcinoembryonic antigen (CEA) level. Meanwhile, ctDNA identified new mutations, including ALK G1202R, I1171N, ALK-ENC1, EML4-ALK, and TP53. PD was verified at follow-up on September 2021, and crizotinib treatment was started. However, severe gastrointestinal effects and hypodynamia prevented the continuation of crizotinib treatment, and ensartinib was chosen for subsequent therapy. After 5 months, he suffered from nausea, vomiting, and pain in the lower back and lower extremities. Chest CT and increased blood tumor markers revealed PD (**Figure 2E**). Brain magnetic resonance imaging (MRI) showed no disease progression. In February 2022, identifying the same driver genes prompted the treatment with lorlatinib, and the best response was PR. After treatment, his pain was significantly alleviated, so he refused to undergo another MRI. At the last follow-up date in December, clinical benefit is still ongoing (**Figure 2F**).

## 3 Discussion

To our knowledge, this is the first report in NSCLC that acquired ALK I1171N and G1202R co-mutation-mediated resistance to ensartinib but responded well to lorlatinib. At the first diagnosis after recurrence, NGS testing on the biopsy sample identified no mutations in EGFR, ALK, or ROS1, which promoted the application of toripalimab. However, the rapid deterioration of the condition prompted double checking of the mutation landscape, and the acquired ALK rearrangement was found in ctDNA, which induced treatment regimen change. It has been reported that ALK-positive NSCLC has a highly aggressive clinical behavior in contrast with wild-type patients (3, 7, 9). Patients with ALK fusions tend to be not sensitive to immune checkpoint inhibitors (10). The resistance to toripalimab increased the likelihood that the driver gene existed. The clinical management of this patient highlights the importance of dynamic monitoring of ctDNA for patients, especially those whose health condition deteriorated rapidly.

Ensartinib is a second-generation ALK inhibitor, achieving a response rate (RR) of 60% and a median PFS of 9.2 months. In ALK TKI-naive patients, RR was 80%, and median PFS was 26.2 months, while in patients with prior TKI treatment, RR was 69%, and median PFS was 9.0 months (11). In this case, the patient experienced a PR and got a PFS of only 5 months. In previous studies, ALK G1202R has been shown to be resistant to ensartinib in patients with lung cancer (12, 13), while I1171N was sensitive to ensartinib in cell lines (14). Here, we found that co-mutation of ALK I1171N and G1202R was resistant to ensartinib.

Lorlatinib is a third-generation ALK inhibitor exploited to penetrate the blood–brain barrier and overcome ALK resistance mutations (15). After PD on ensartinib, lorlatinib was given because of its accessibility in mainland China. The phase 2 study of lorlatinib in patients with ALK-positive lung cancer showed that 87.0% of patients with measurable baseline CNS lesions achieved an intracranial response. It has been well-documented that acquired ALK G1202R is sensitive to lorlatinib (13, 15–17). In addition, I1171N was recently found to be more sensitive to lorlatinib than to crizotinib and alectinib in the preclinical study (18). Significantly, a case reported that the multiple ALK mutation, including I1171N, L1196M, and G1202R, mediated the resistance to lorlatinib in malignant pleural mesothelioma. However, in this case, we reported that the co-mutation of I1171N and G1202R was sensitive to lorlatinib (19). Our case with acquired multiple mutations respond differently to different ALK-TKIs, contributing to the understanding of complex ALK-TKI resistance mechanisms.

## 4 Conclusions

As reported in previous clinical trials, ALK fusions were sensitive to ALK inhibitors, especially EML4-ALK fusion. However, different rare ALK fusions respond differently to various generation ALK inhibitors. Here, we reported a patient harboring multiple ALK fusions, including ALK G1202R, I1171N, and EML4-ALK, who was sensitive to lorlatinib. This case provides evidence that this complex ALK fusion combination is sensitive to the third-generation ALK inhibitor.

## Data availability statement

The raw data supporting the conclusions of this article will be made available by the authors, without undue reservation.

## Ethics statement

The studies involving human participants were reviewed and approved by Ethics Committee of Hangzhou Traditional Chinese Medicine Hospital. The patients/participants provided their written informed consent to participate in this study. Written informed consent was obtained from the individual(s) for the publication of any potentially identifiable images or data included in this article.

## Author contributions

ZY and JG followed patients and collected patient data. JG wrote the initial draft. All authors contributed to the article and approved the submitted version.
